# Using High-Throughput Phenotyping to Explore Growth Responses to Mycorrhizal Fungi and Zinc in Three Plant Species

**DOI:** 10.34133/2019/5893953

**Published:** 2019-03-25

**Authors:** S. J. Watts-Williams, N. Jewell, C. Brien, B. Berger, T. Garnett, T. R. Cavagnaro

**Affiliations:** ^1^The School of Agriculture, Food and Wine, and The Waite Research Institute, The University of Adelaide, PMB 1, Glen Osmond, SA 5064, Australia; ^2^Australian Research Council Centre of Excellence in Plant Energy Biology, The University of Adelaide, Glen Osmond, SA, Australia; ^3^Australian Plant Phenomics Facility, The Plant Accelerator, The University of Adelaide, PMB1, Glen Osmond, SA 5064, Australia

## Abstract

There are many reported benefits to plants of arbuscular mycorrhizal fungi (AMF), including positive plant biomass responses; however, AMF can also induce biomass depressions in plants, and this response receives little attention in the literature. High-throughput phenotyping (HTP) technology permits repeated measures of an individual plant's aboveground biomass. We examined the effect on AMF inoculation on the shoot biomass of three contrasting plant species: a vegetable crop (tomato), a cereal crop (barley), and a pasture legume (Medicago). We also considered the interaction of mycorrhizal growth responses with plant-available soil zinc (Zn) and phosphorus (P) concentrations. The appearance of a depression in shoot biomass due to inoculation with AMF occurred at different times for each plant species; depressions appeared earliest in tomato, then Medicago, and then barley. The usually positive-responding Medicago plants were not responsive at the high level of soil available P used. Mycorrhizal growth responsiveness in all three species was also highly interactive with soil Zn supply; tomato growth responded negatively to AMF inoculation in all soil Zn treatments except the toxic soil Zn treatment, where it responded positively. Our results illustrate how context-dependent mycorrhizal growth responses are and the value of HTP approaches to exploring the complexity of mycorrhizal responses.

## 1. Introduction

Although arbuscular mycorrhizal fungi (AMF) are known to play a major role in the uptake of plant nutrients, the impacts of forming arbuscular mycorrhizal (AM) associations on plant growth can vary widely [1]. Positive growth responses to AM colonisation are the most often reported plant responses in the literature [2, 3]; however, the so-called ‘mycorrhizal growth depressions', whereby a mycorrhizal plant accumulates less biomass than a nonmycorrhizal control plant, are receiving increasing attention [4–6]. The mechanisms behind AMF-induced plant growth depressions still remain unclear, but it is plausible that they are related to an imbalance in the trade of resources between host plant and fungus (i.e., C from plant and P from the fungus) [5]. Understanding when AM associations have a negative impact is important for optimising nutrition management practices for major crops such as wheat and barley.

To better understand AM functioning and the role of the plant-fungus interaction more broadly, we need to understand the causes and development of mycorrhizal growth depressions, and the interacting factors. For example, some plant species can be generally categorised as displaying ‘responsive' or ‘nonresponsive' growth phenotypes to AM fungal inoculation [7]. Commonly studied plant species such as maize (*Zea mays*), leek (*Allium porrum*), and Medicago (*Medicago truncatula*) often display marked positive growth responses when colonised by AMF, particularly under soil nutrient deficient conditions [8, 9]. However, there has also been some focus in the literature on plant species such as wheat (*Triticum aestivum*) and barley (*Hordeum vulgare*) being nonresponsive or even negatively responsive to AMF inoculation [10–13]. To further complicate matters, growth responses are not consistent between different genotypes of the same species of plant [13, 14], nor between different species (or isolates) of colonising AMF, on the same plant genotype (summarised in [12]).

The response of plant biomass to mycorrhizal inoculation is also dependent on soil nutrient availability, particularly the amount of plant-available phosphorus (P) and zinc (Zn) in the soil [15]. High soil P concentration generally suppresses the colonisation of roots by AMF [16], and in addition, the interplay between the direct and mycorrhizal pathways of plant P uptake is highly dependent on soil P availability [17, 18]. Furthermore, available soil P is highly interactive with soil Zn and impacts upon the uptake of both nutrients [15, 19–21]. Plants that are subjected to soil Zn stress, either through deficient or toxic concentrations of plant-available Zn in the soil, generally benefit from being colonised by mycorrhizal fungi [22]. This is because when soil Zn is deficient, AMF can acquire Zn from the soil outside of the root's depletion zone and transfer this to the plant, thereby reducing the stress. In toxic Zn conditions, AMF have the ability to prevent the plant from accumulating toxic concentrations of Zn in their tissues [23, 24], although the protective mechanisms remain unclear. It is because of these complex and varied responses to soil Zn availability that it is important to study plant responses to mycorrhizal inoculation under a range of Zn treatments.

A better understanding of the growth response to AM can be achieved through time series data. Such data sets can be laborious and costly to generate, as they usually rely on multiple harvests. High-throughput phenotyping (HTP) platforms have more recently provided a solution for this [25] and reduce numerous confounding variables by tracking the growth of the same plant through its life. Previous experiments utilising HTP have proved valuable for studies of genetic variation in traits in chickpea, barley, wheat, and rice [26–29].

Here we present results of two glasshouse experiments exploring shoot mycorrhizal responsiveness using a HTP phenotyping platform. In the first experiment we grew two plant species that are known to generally have neutral (tomato) or negative (barley) growth responses when inoculated with the AMF* Rhizophagus irregularis*. We quantified their growth over time to explore the different responsiveness phenotypes at different soil Zn availabilities. In the second experiment we grew Medicago at high soil P and a range of soil Zn availabilities to quantify the growth over time of a typically positive-responsive species and explore the interaction with soil P and Zn availability. Our aim was to measure shoot growth over time to pinpoint the appearance of a growth depression in order to gain a deeper perspective on AMF effects on plant growth.

## 2. Materials and Methods

### 2.1. Experiment 1: Tomato and Barley

Each plastic pot (180 mm height, 90 mm width and length, and 0.97 L volume) contained 1.4 kg of soil/fine sand mixture (10:90) that had been mixed thoroughly with 140 g (constituted 10% soil weight) of either an AMF (*Rhizophagus irregularis* WFVAM10, synonymous with DAOM 181602, an earlier voucher number for DAOM 197198, formerly named* Glomus intraradices*) inoculum, or a control inoculum and mixed thoroughly. The* R. irregularis* inoculum was added as a mix of dry soil, fungal spores and external hyphae, and root fragments of Marigold (*Tagetes patula*) pot cultures produced on-site. The control, a mock inoculum, was a mixture of dry soil and root fragments of Marigold pots that had not been inoculated with AMF. The soil originated from the Waite Arboretum, located at the University of Adelaide, Australia (coordinates: 34.9670°S, 138.6360°E); it was an Urrbrae red-brown earth [30] and had (KCl-extractable) NO_3−_ (nitrate) and NH^+^_4_ (ammonium) concentrations of 11 and 55 mg N kg^−1^, respectively, a (Colwell; estimated plant-available) P concentration of 32 mg P kg^−1^, and a (DTPA-extractable; estimated plant-available) Zn concentration of 15 mg Zn kg^−1^. Before being autoclaved twice, the soil was sieved to <2 mm to homogenise and dried at 60°C, before being mixed in proportion with the twice-autoclaved and dried sand. Following mixing with sand, the Colwell P concentration of the soil/sand mix was reduced to 3.4 mg P kg^−1^.

In order to establish the soil Zn treatments, soil was amended with Zn sulphate (as ZnSO_4_.7H_2_O) to 10, 40, and 90 mg Zn kg^−1^, which consequently resulted in the DTPA-extractable Zn concentrations of 8.9, 32, and 61 mg Zn kg^−1^. These three Zn treatments are referred to as Zn 10, Zn 40, and Zn 90, respectively. The Zn 0 treatment soil was unamended soil/sand mix, and had a DTPA-extractable Zn concentration of 1.7 mg Zn kg^−1^.

Seeds of* Hordeum vulgare* (barley) cv. Compass and* Solanum lycopersicum* (tomato) cv. Rio Grande 76R were surface-sterilised in 10% bleach solution while being shaken for 10 mins and then rinsed in reverse osmosis (RO) water, before finally being shaken for ten more minutes in RO water to clean thoroughly of bleach. Petri dishes containing moist filter paper were used as the substrate to germinate the seeds; once rinsed seeds were placed gently onto the filter paper, Petri dishes were sealed and incubated at 25°C for three days in the dark. Plates of germinating seeds were placed in the light at room temperature for 24 h, before the seeds were sown directly into the prepared pots at a depth of 20 mm. Two pregerminated seeds were planted per pot and were thinned to one plant per pot after 7 days. There were four biological replicates per treatment. From the second week, all plants received 10 mL of a half-strength modified Long-Ashton solution, omitting P and Zn (following [31]), on a weekly basis. See below for growth and phenotyping conditions.

### 2.2. Experiment 2: Medicago

The soil, sand, and inoculum were prepared as for Expt. 1, except that the ratio of soil to sand was lowered to 5:95 for this experiment, to further reduce the baseline Zn concentration. Due to the low proportion of soil mixed in with the sand and the need to increase plant-available P for the specific objective of this experiment, supplemental P was added to the soil as CaHPO_4_ (anhydrous) at a rate of 10 mg P kg^−1^ which increased the final soil Colwell P concentration to 20 mg P kg^−1^.

The soil Zn addition treatments were once again established by applying Zn sulphate at the rates of 2, 5, 10, 20, and 30 mg Zn kg^−1^, which resulted in five soil DTPA-extractable Zn concentrations: 1.7, 4.0, 6.5, 15.0, and 23.0 mg Zn kg^−1^, now known as Zn 2, Zn 5, Zn 10, Zn 20, and Zn 30, respectively. The Zn 0 treatment soil was not subject to any Zn addition and had a DTPA-extractable Zn concentration of 0.3 mg Zn kg^−1^.

Seeds of* Medicago truncatula* cv. Jemalong A17 were surface-sterilised by immersion in 10% bleach solution, then shaken for ten minutes, and then rinsed well before being shaken in RO water for ten mins to rinse thoroughly. The seeds were blotted well with paper towel to dry; then seed coats were scarified using fine sandpaper, before being plated onto moist filter paper in a Petri dish, sealed, and kept at 4°C in the dark for five days. Following the cold treatment, the seeds were transferred to 25°C but remained covered for two days and then uncovered and left at room temperature for two days. Once the germinated* M. truncatula* seedlings displayed expanded and green cotyledons, they were gently planted into the prepared soil in pots (two plants per pot) and thinned to one seedling per pot after seven days. There were six biological replicates per treatment. As for Expt. 1, from the second week, all plants received 10 mL of half-strength modified Long-Ashton solution, omitting P and Zn, on a weekly basis. In addition, the Medicago plants each received a total of 100 mg N as NH_4_NO_3_ over the course of the experiment (30, 25, 25, and 20 mg N plant^−1^ at 4, 20, 34, and 48 DAP, respectively) in order to suppress associations with rhizobial bacteria. See below for growth and phenotyping conditions.

### 2.3. Plant Growth, Phenotyping, Harvesting, and Sample Analysis

The tomato and barley plants were grown between February and April 2017 (Austral Summer/Autumn), and the Medicago plants between October and November 2017 (Austral Spring) in a glasshouse within The Plant Accelerator, Australian Plant Phenomics Facility, located at the University of Adelaide, Waite campus, Australia. Mean temperature for Expt. 1 was 27°C/17°C day/night with mean PAR of 675 *μ*mol m^−2^ s^−1^ at midday. For Expt. 2, mean temperature was 26°C/16°C day/night with mean PAR of 834 *μ*mol m^−2^ s^−1^ at midday. For the first 16 days (tomato and barley) or 21 days (Medicago), plants were grown on benches. Following this, all plants were moved onto the phenotyping cart system in the same glasshouse and were then imaged [28] and watered gravimetrically (to 10% soil weight) by the automatic system on a daily basis until harvest.

For barley and tomato, it should be noted that there was an unscheduled three-day interruption to watering (DAP 39-41 inclusive). This had an appreciable (negative) effect on the observed growth rates past DAP 43, particularly for plants in the tomato mock-inoculated control group. This is to be expected, given that the mock-inoculated plants were generally larger in size (based on projected shoot area [PSA] at DAP 27, 33, and 39) up to the onset of the interruption. Thus, we have statistically analysed PSA after DAP 43, but not presented it in the Figures.

After 53 days after transplantation, both the barley and tomato plants were destructively harvested, while both species were still in the vegetative phase of growth. The Medicago plants were harvested 54 days after transplantation, at the onset of flowering. Both experiments were harvested as follows: RO water was used to wash roots clean of any attached soil, shoots were separated from roots at the soil surface, and the fresh mass of both shoots and roots, respectively, weighed. A weighed subsample consisting of ~200mg fresh root biomass was placed into 50 % EtOH to be fixed prior to staining. Any remaining root biomass and the total shoot biomass were then placed in a drying oven at 60°C for at least 48 hours before measures of dry mass were taken. To prepare for acid digestion, the shoot biomass was homogenised before a weighed subsample of ~250mg was digested using a 4:1 (*v/v*) mix of nitric acid and hydrogen peroxide. Plant digests were subsequently diluted with RO water and then analysed for elemental concentrations by ICP-AES. Fresh roots fixed in 50% EtOH were rinsed and then cleared by being submerged in 10% KOH solution at 25°C for seven days. Once sufficiently cleared, the roots were rinsed thoroughly with RO water and then stained for 10 minutes in 5% ink in vinegar (modified from [32]) solution heated to 60°C. Following staining, roots were destained in acidified water for at least 24 hours and then stored in 50% glycerol solution. The gridline intersect method Giovannetti and Mosse [33] was used to determine the percentage root length colonised by AMF for each sample.

### 2.4. Statistical Design

#### 2.4.1. Expt. 1: Tomato and Barley

The design for each experiment was a randomised complete block design with four replicates of the eight treatments. Two replicates are located in each of two lanes in the automated platform. The design was randomised using dae [34], a package for the R statistical computing environment [35]. Imaging was carried out daily from DAP 17 to DAP 51. From these images the PSA of the plant, as viewed using an RGB camera, was obtained; it is the sum of the areas as measured (in kilopixels) from two side views at an angular separation of 90 degrees and a view from above [28].

#### 2.4.2. Expt. 2: Medicago

There were two mycorrhizal inoculation treatments (no mycorrhizal inoculation (-) and mycorrhizal inoculation (+)) and six levels of Zinc (0, 2, 5, 10, 20, and 30 mg Zn kg^−1^ soil), resulting in 2×6=12 treatment groups. The experiment utilised a split-plot design with six replicates of the 12 treatments, with each replicate occupying half of one lane on the automated platform. The design was randomised using dae [34], a package for the R statistical computing environment [35]. Imaging was carried out daily from DAP 22 to DAP 53. From these images the PSA of the plant was determined as described above, except that, following an upgrade of the camera system since Expt. 1, the Medicago analysis was based on 4 camera views: one top, one side, and two oblique.

### 2.5. Data Preparation

The imaging data was prepared using the package imageData [36] for the R statistical computing environment [35]. Spline smoothing was applied to the PSA growth curve of each plant, yielding smoothed projected shoot area (sPSA). Initially, probeDF from imageData was used to examine subjectively the degree of smoothing achieved for sPSA. The values of six degrees (mild smoothing) for barley and tomato and five degrees (stronger smoothing) for Medicago were chosen for this trait, as they were judged to give the most satisfactory result for each experiment, respectively.

After examination of the plots for the smoothed traits sPSA, it was decided to statistically examine sPSA on the following dates: 27, 33, 39, and 43 DAP (tomato and barley) and 27, 33, 39, 43, and 51 DAP (Medicago).

### 2.6. Statistical Analysis

To produce phenotypic means a mixed-model analysis was performed for each trait from each experiment using the ASReml-R [37] and asremlPlus [38] packages for the R statistical computing environment [35]. The maximal mixed-model for this analysis was of the form

(1) $$\begin{array}{c} y=X\beta +Zu+e, \end{array}$$

where **y** is the response vector of values for the trait being analysed; **β** is the vector of fixed effects; **u** is the vector of random effects; and **e** is the vector of residual effects. **X** and **Z** are the design matrices corresponding to **β** and **u**, respectively. The fixed-effect vector **β** is partitioned as $\left[\begin{array}{ccccc} \mu & {\beta^{\prime }}_{R}^{} & {\beta^{\prime }}_{M}^{} & {\beta^{\prime }}_{Z}^{} & {\beta^{\prime }}_{M:Z}^{} \end{array}\right]$, where *μ* is the overall mean and the **β** subvectors correspond to the respective effects of Replicates, Mycorrhiza, Zinc, and Mycorrhiza × Zinc interaction. Thus, **β** subvectors 2-4 were of intrinsic interest, while the first subvector removed the effects of spatial variation within the automated platform. The random-effects vector **u** applied only to the Medicago experiment, where it captured the random variation associated with differences between main-plots within replicates. The residual effects **e** were assumed to be normally distributed with zero mean and variance *σ*^2^, except for the barley experiment where unequal variances *σ*_−_^2^ and *σ*_+_^2^ were assumed for the two Mycorrhiza levels. To test whether *σ*_−_^2^ and *σ*_+_^2^ were significantly different, both differential-variance and equal-variance models were fitted, and the results are compared using a REML ratio test.

For all plants, Wald F-tests were conducted for an interaction between* Mycorrhiza* and* Zinc* for sPSA and, if the interaction was not significant, for their main effects. The predicted means were obtained for the combinations of* Mycorrhiza* and* Zinc* levels.

Finally, for all three plant species, two-way ANOVA were conducted on the final harvest data (biomass; nutrient concentrations) with* Mycorrhiza* and* Zinc* the factors. Where there was a significant interaction term, or in the absence of a significant interaction, a significant main effect, a Tukey's HSD* post hoc* test was performed. Dry weight and nutrient concentration figures were generated using the ggplot2 package for the R statistical computing environment [35]. Principal components analysis (PCA) was used to analyse the data on shoot elemental concentrations (P, K, Ca, Mg, S, Mn, Fe, Cu, and Zn) and biplots were constructed to visualise the shoot “ionome”, with* Mycorrhiza* included as supplemental variable in the analysis. The analyses of harvest time point data were conducted using JMP (version 14.0.0).

## 3. Results

### 3.1. Mycorrhizal Colonisation

**Figure 1:**
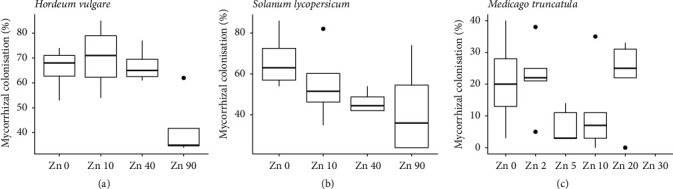
Mycorrhizal colonisation at harvest of barley (a), tomato (b), and Medicago (c) inoculated with the AMF* R. irregularis *and grown at four (a, b) or six (c) different soil Zn concentrations ranging from no addition of Zn (Zn 0) to high soil Zn addition. See Supplementary Table 1 for details of ANOVA results. Intervals on each box represent the minimum, Q1, median, Q3, and maximum values and any outlier values (dots),* n*=4 (a, b);* n*=6 (c).

Both the barley and tomato roots were well colonised by the AMF in inoculated treatments, for all Zn addition treatments. Mean values of mycorrhizal colonisation in barley roots ranged from 41.5 to 69.4 % (Figure 1(a)), and, similarly in tomato, colonisation of roots ranged from 42.5 to 70.7 % (Figure 1(b)). However, mycorrhizal colonisation was reduced by increasing soil Zn concentration in the barley plants only (Supplementary Table 1). The Medicago plants were far less colonised by AMF than the other plant species, with values between 7.8 and 27.8% root length colonised (Figure 1(c)). The roots of the mock-inoculated plants of all three species had no colonisation by AMF (data not shown). Furthermore, the Medicago plants had no nodules, indicating that suppression of the rhizobial symbiosis by N supplementation was effective.

### 3.2. Plant Biomass and Nutrition at Harvest

For barley, the shoot and root dry weights at harvest of the noninoculated plants were larger than those of the mycorrhizal plants and pooling* Zinc* treatments (Supplementary Table 1; Supplementary Table 2). There was a main effect of* Zinc* on root dry weight, whereby Zn 0 roots were larger than Zn 90 roots and also on root to shoot ratios, whereby the Zn 0 plants had a greater mean root to shoot ratio than the Zn 40 and Zn 90 plants. For tomato, shoot dry weights were not different between inoculated and noninoculated plants except at the highest soil Zn concentration (Zn 90), where the shoot dry weight of the inoculated plants was higher than the noninoculated plants (Supplementary Table 2). Conversely, the tomato root dry weights were greater in the noninoculated plants in the Zn 0 treatment, and the root to shoot ratios was mostly consistent, except that they were higher in the noninoculated Zn 90 plants than in almost every other treatment, except the noninoculated Zn 0 plants. For Medicago, there were main effects of* Mycorrhiza*, with both the shoot and root dry weights of noninoculated plants being higher than those of their inoculated counterparts when pooled over* Zinc* treatments. Further, there were main effects of* Zinc* (i.e., pooling* Mycorrhiza* treatments), whereby plants in the Zn 5 treatment had greater shoot biomass than those in Zn 20 or Zn 30, and the plants in the Zn 30 treatment had smaller root biomass and root to shoot ratio than all the other Zn treatments.

**Figure 2:**
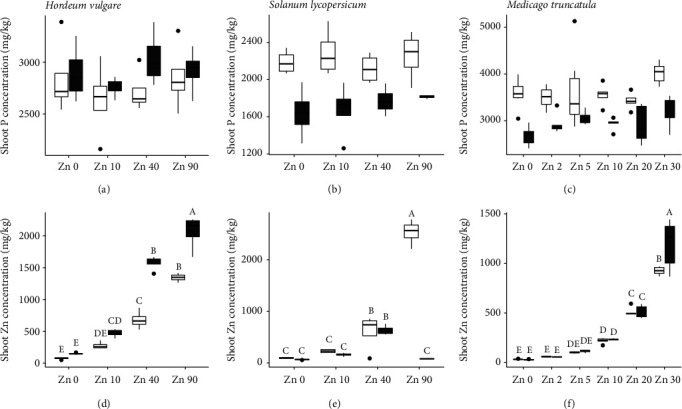
Shoot phosphorus concentrations (mg kg^−1^) at harvest of barley (a), tomato (b), and Medicago (c) and shoot zinc concentrations (mg kg^−1^) at harvest of barley (d), tomato (e), and Medicago (f) inoculated with the AMF* R. irregularis *(grey) or mock-inoculated (white) and grown at four (a, b, d, e) or six (c,f) different soil Zn concentrations ranging from no addition of Zn (Zn 0) to high soil Zn addition. Means followed by the same letter were not significantly different at the P<0.05 level (Tukey's HSD); see Supplementary Table 1 for details of ANOVA results.* n*=4 (a, b, d, e),* n*=6 (c, f). Note: y-axis does not begin at zero for (a), (b), and (c).

While there was no effect of* Mycorrhiza* or* Zinc* treatments on the shoot P concentration of barley, there was a main effect for* Mycorrhiza* for both the tomato and the Medicago plants (Supplementary Table 1; Figures 2(a)–2(c)); for both plant species, the noninoculated plants had greater shoot P concentrations than the inoculated plants, pooling* Zinc*. For all three plant species, there was a significant interaction between* Mycorrhiza* and* Zinc* when shoot Zn concentration was considered. For barley, shoot Zn concentrations increased with increasing soil Zn concentration in both the inoculated and noninoculated plants; in the Zn 40 and Zn 90 treatments, the inoculated plants had higher shoot Zn concentrations than the noninoculated plants (Figure 2(d)). In the tomato plants, shoot Zn concentration increased between Zn 0 and Zn 90 in the noninoculated plants, whereas in the inoculated plants, shoot Zn concentration increased from Zn 0 to Zn 40 and then decreased at Zn 90 (Figure 2(e)). This meant that at Zn 90, the noninoculated plants had higher Zn concentrations than the inoculated ones. The Medicago plants followed a similar trend to barley, where shoot Zn concentrations increased steadily in both inoculated and noninoculated plants between Zn 0 and Zn 30, but at Zn 30, the inoculated plants had higher Zn concentrations than the noninoculated plants (Figure 2(f)).

For barley, principal component 1 (PC1) explained 46.7% of the variation in the shoot ion data and was driven by shoot S and K concentrations (Supplementary Figure 1a). PC2 explained a further 20.5% and was driven in the positive direction by Mn concentration and in the negative direction by Mg concentration. When* Mycorrhiza* was included as a supplementary variable, the noninoculated plants were separated from the inoculated plants primarily on PC2.

Similarly, for tomato PC1 explained 64.1% of the variation and was driven by increasing shoot Ca, S, Zn, and K concentrations (Supplementary Figure 1b). PC2 explained a further 21.6% and was driven in the positive direction by P concentrations and in the negative direction by Mg concentration. The inoculated and noninoculated plants again separated out primarily on PC2. Furthermore, the noninoculated Zn 90 plants separated out from all the other plants on PC1 due to higher concentrations of elements including Zn. In contrast, the Medicago plants did not separate out by inoculation treatment (Supplementary Figure 1c). PC1 explained 45.1% of the variation and was driven by increasing shoot concentrations of S, Mn, Zn, and K. The ionomes of the highest Zn treatment (Zn 30) plants were mostly driven by PC1. PC2 explained a further 21.7% of variation and was driven by increasing Cu, Ca, and Mg concentrations.

### 3.3. Plant Growth over Time: Shoot Phenotyping

**Figure 3:**
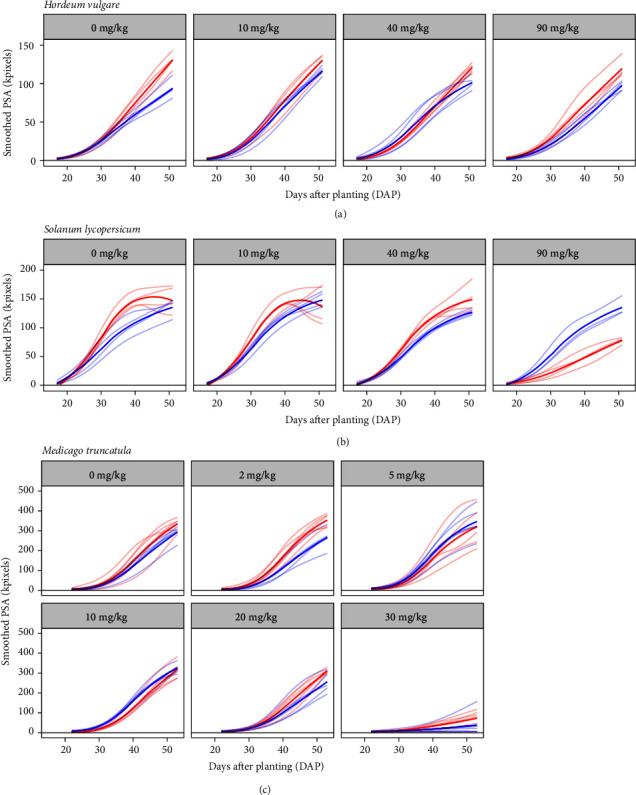
Smoothed PSA over time grouped by Zn addition treatment in barley (a), tomato (b), and Medicago (c) plants inoculated with the AMF* R. irregularis *(blue) or mock-inoculated (red) and grown at four (a, b) or six (c) different soil Zn concentrations ranging from no addition of Zn (Zn 0) to high soil Zn addition. On each panel, the darker lines represent the loess mean PSA of replicates within a treatment, while lighter lines correspond to individual replicates.

Smoothed values of projected shoot area (sPSA) were used to monitor growth over time. To give an overview of growth over the course of the experiments, descriptive plots based on daily sPSA values were constructed for each plant species, smoothed across treatment replicates, and split by Zn treatment (Figures 3(a)–3(c)), or by Mycorrhiza treatment (Supplementary Figure 2a-c). For barley the appearance of a growth depression in the inoculated plants is dependent on soil Zn concentration (Figure 3(a)), starting much earlier in the Zn 90 plants than the Zn 10 plants. For tomato plants, the rapid and large positive response to AMF inoculation in the Zn 90 plants contrasts with the slower-appearing negative responses in the other Zn treatments (Figure 3(b)). Meanwhile, the negative effect of high soil Zn concentration (Zn 30) on shoot biomass was highly pronounced in the Medicago plants (Figure 3(c)).

**Figure 4:**
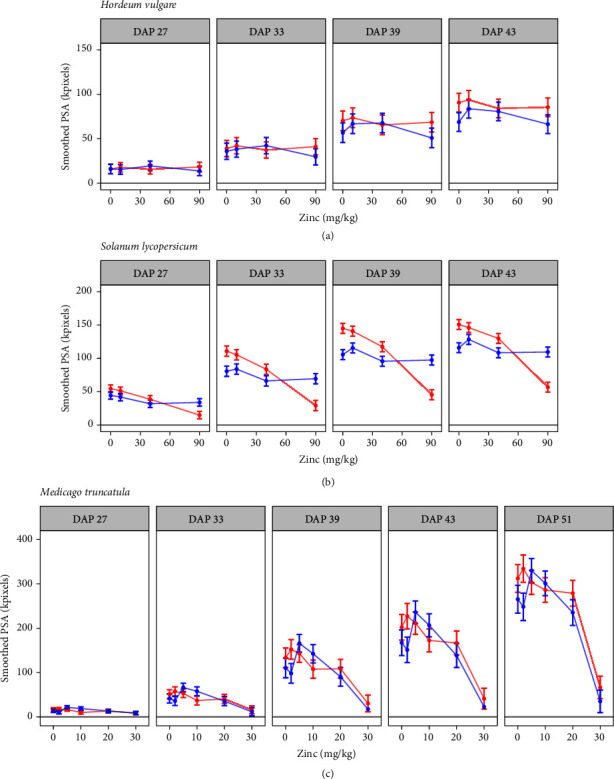
Predicted smoothed PSA for all 8 combinations of* Mycorrhiza* and* Zinc* in barley (a), tomato (b), and all 12 combinations in Medicago (c) inoculated with the AMF* R. irregularis *(blue) or mock-inoculated (red) and grown at four (a, b) or six (c) different soil Zn concentrations ranging from no addition of Zn (Zn 0) to high soil Zn addition, at four or five time points during growing. These predictions are based on the full interaction model, notwithstanding the fact that interaction fails to be statistically significant for barley at any time point. Error bars for barley are 95% confidence intervals. Error bars for tomato and Medicago are half of the least significant difference (LSD), meaning that two predictions are significantly different if and only if their error bars do not overlap.

For barley, the main effect of* Mycorrhiza* on sPSA was apparent at days 39 and 43, such that the inoculated plants had lower predicted values than the noninoculated plants (Supplementary Table 3; Supplementary Figure 3), with no interaction between* Mycorrhiza* and* Zinc* found (Figure 4(a)). For tomato, a statistically significant interaction between* Mycorrhiza* and* Zinc* was observed for all five time points (Supplementary Table 3; Figure 4(b)). For Medicago, a statistical interaction between* Mycorrhiza* and* Zinc* was observed for PSA, for days 33, 39, and 43 only (Supplementary Table 3; Figure 4(c)). The analyses of sPSA revealed that a growth depression (i.e., mock-inoculated plants larger than mycorrhizal plants in any given Zn treatment) appeared at different times for each plant species, manifesting first in tomato (33 DAP) at Zn 0, Zn 10, and Zn 40, followed by Medicago (39 DAP) at Zn 2 and barley (43 DAP) at Zn 0.

## 4. Discussion

High-throughput phenotyping (HTP) technology permits nondestructive, daily measurements of plant aboveground biomass, allowing for repeated biomass measures of the same plant over its life until harvest, thereby reducing the error and labour that is involved with multiple destructive harvests of different plants. Plants respond to and are colonised by AMF at different times and intensities, meaning the symbiosis develops over time differently for each individual plant-fungus association. Therefore, the study of biomass responses to AM over the course of a plant's development is well suited to HTP. In what we believe to be the first application of HTP of this type in studies of AM, we have examined the effect of AMF inoculation and varying soil Zn concentrations on the growth of three contrasting species: a vegetable crop, cereal crop, and pasture legume. We found that mycorrhizal growth responsiveness at final harvest differed between plant species, and that it was modulated by soil Zn supply. Moreover, we found that the appearance of a Mycorrhiza-induced depression in shoot growth occurred at different times after planting for each plant species.

### 4.1. Mycorrhizal Inoculation Can Induce Growth Depressions

There is a dearth of studies that explore negative and neutral plant growth responses to mycorrhizal colonisation, especially relative to positive growth responses; this is perhaps due to publication bias. However, it is increasingly understood that a thorough understanding of the mycorrhizal symbiosis depends upon a comprehensive view of the range of responses (e.g., beyond growth, plant nutrition should also be considered) that diverse plant species have to different species and isolates of AMF. Growth data over time allows us to pinpoint the age of the plant where a growth depression begins and allows us to speculate on when a plant has become colonised enough for a difference in biomass accumulation to manifest.

In the present study, all three plant species experienced shoot growth depressions as a result of being colonised by the AMF* R. irregularis*. However, the appearance of a detectable shoot growth depression occurred at a different age for each species; the tomato plants were the first species to display a visible growth depression (33 DAP), followed by Medicago (39 DAP) and barley (43 DAP). The precise mechanism/s behind mycorrhiza-induced growth depressions remain to be uncovered, although there are a number of hypotheses that have been proposed (see Jin, Wang et al. [5] for recent review), some of which we explore here.

One hypothesis to explain mycorrhizal growth depressions is that of ‘unbalanced C-for-nutrient trade' between the plant and AMF, whereby the colonising fungi cause a C-drain on the host plant by receiving plant carbon resources and not proportionally reciprocating with P transported to the plant. In this study, the barley plants had the highest percentage of root length colonised by AMF structures (~68%) out of the three, yet the slowest and smallest negative mycorrhizal shoot response. However, Grace, Cotsaftis et al. [11] discovered that the mycorrhizal growth depression observed in their barley plants could not be accounted for simply by unequal C-P trade, as the contribution to plant P via the mycorrhizal pathway was substantial, and proportional to root length colonised. Similar findings have also been reported for wheat [39, 40]. Further, Grace, Cotsaftis et al. [11] postulated that, instead of an AMF-induced C-drain, the direct pathway of P uptake was downregulated and this caused a shortfall in total plant P supply, resulting in a growth depression; this hypothesis may explain the shoot growth depressions for tomato and Medicago here, where shoot P concentration was lower than that of the noninoculated plants, but is not consistent with the barley results here, where shoot P concentrations were matched between inoculation treatments. Clearly, further investigation is needed to elicit the mechanism behind AM-induced growth depressions in barley, potentially including radioisotope tracing studies.

Regarding the tomato plants, it appears that AMF colonisation of the roots was rapid, due to the appearance of a growth depression by 33 DAP. Rapid colonisation could have imposed stress on the plant's C resources while it was still establishing sufficient photosynthetic area to maintain essential processes. Using the same tomato cultivar, Smith et al. [41] also observed a large growth depression with AMF inoculation (*Glomus intraradices*); importantly, they also showed that despite the growth depression, by 5 weeks of growth, almost 100% of the tomato's P was derived via the mycorrhizal pathway of uptake. For all three plant species in our study, once a growth depression appeared, it persisted until harvest. However, the magnitude of the growth depression did not increase as the plants grew, which suggests that if there was an initial cost to forming AM, it did not increase over the life of the plant. This result deserves further investigation into the effects of AMF on C-P trade over the life of the plant.

### 4.2. Plant Responsiveness Is Influenced by the Soil Zn and P Concentration

#### 4.2.1. Soil Zinc

For both the barley and Medicago plants, mycorrhizal growth depressions only appeared in one out of the four and six Zn treatments, respectively; growth was otherwise matched between the mycorrhizal and nonmycorrhizal plants (a neutral mycorrhizal response). In contrast, the tomato plants showed strong responses in either a negative or positive direction to AMF in all the Zn treatments [15]. The greatest depression in growth was observed at Zn-deficient soil concentrations (Zn 0) for both barley and tomato. The limitation of Zn in this treatment may be exacerbated by the uptake of P by the mycorrhizal pathway in such a way that the plant is not P-limited, but instead Zn-limited, and this appeared as a growth depression in response to AMF inoculation.

However, when Zn in the soil reached toxic concentrations for tomato (Zn 90), plant biomass responded very positively to mycorrhizal inoculation; shoot mass was 2.5 times greater by the time of harvest. Furthermore, this positive growth response at high Zn appeared even earlier than the growth depressions in the other Zn treatments, by 27 DAP. This ‘switch' in growth response from negative to positive with increased Zn concentration may be due to the ‘protective effect' of AMF at high soil concentrations of Zn [24], whereby the mycorrhizal plant is more tolerant of high soil Zn. In contrast, there was no evidence of the AM protective effect in the highest soil Zn concentration for barley or Medicago, although it has been observed in previous work [42]. For barley this may be because it is a species that is relatively tolerant of a wide range of soil Zn concentrations, both low and high, as in other studies using this cultivar [43] and other cultivars [44, 45]. Medicago is discussed in the context of plant-available soil P below.

#### 4.2.2. Soil Phosphorus

In a previous study using the same soil, AMF inocula, and range of soil Zn treatments, we observed highly positive growth responses in* Medicago truncatula* A17 both at low and high soil Zn concentrations [42]. Here, the growth of the Medicago plants was either neutral or negative in response to AMF inoculation, depending on the soil Zn concentration. The P concentration of the soil in the present study (20 mg P kg^−1^) was double that in Watts-Williams, Tyerman et al. [42] (9.6 mg P kg^−1^). In a previous study using the same soil and species of AMF, Facelli et al. [46] also found that Medicago plants experienced a growth depression in response to mycorrhizal inoculation at high soil P, but a positive response at low soil P. Clearly, the available P in the soil is a strong determinant of Medicago growth response to AMF inoculation. One part of the explanation may be that when soil P is highly available, mycorrhizal colonisation is suppressed and the plant does not engage the mycorrhizal pathway of P uptake [47]. Measures of root length colonised were indeed vastly lower in the present study (5-25 %) than in Watts-Williams, Tyerman et al. [42] (75-90%). Another part of the explanation may be that when plant-available soil P is plentiful, the effects of soil Zn toxicity can be avoided by the plant, because the Zn taken up becomes ‘diluted' in plant tissue with increased P uptake and thence biomass [48]. Taken together, we conclude that* Medicago truncatula* did not require the benefits of being colonised by AMF because the soil available P was high, even when soil Zn concentration posed as a stressor through deficiency or toxicity.

### 4.3. The Effect of AMF on Shoot Nutrition Varies with Plant Species

As well as an important determinant of plant biomass accumulation, AMF play a role in nutrient uptake and thus the mineral composition of plants. It is important to highlight that even in cases where a neutral or negative plant growth response is elicited, there may still be benefits to plant nutrition; this point has been demonstrated by the radioisotope P tracing studies mentioned above [11, 41] and also by Zn radioisotope tracing in the 76R tomato cultivar [49].

The shoot ionome [50], that is, the mineral composition of the plant based on nine elements measured by ICP-AES, at harvest differed across the three plant species. The tomato ionome separated by mycorrhizal treatment the most out of the three species; this result agrees with the stark difference in growth between the mycorrhizal treatments over time and across Zn treatments. Clearly, AMF have a striking effect on both the growth and the mineral nutrition of tomato plants [51, 52]. To a lesser degree, this separation between mycorrhizal and nonmycorrhizal plants was also observed in the barley ionome. More specifically, the Zn contents of the mycorrhizal barley plants were greater than that of the nonmycorrhizal plants. This points to the improvement of Zn uptake by AMF, even when growth was either negative or neutral and has been shown previously in this cultivar of barley [43].

### 4.4. Conclusions

Generally, the literature on AM focuses on the benefits to yield and/or nutrition of the host plant. Here we have explored in detail the advent of neutral and negative growth responses to mycorrhizal inoculation in three plant species by measuring their growth over time. The results highlight the strong context dependency of growth responses, particularly in terms of plant species, time point, and soil nutrient (P and Zn) availability. No doubt many other factors such as AMF species, water availability, and temperature are also important factors for determining the response of plants to AMF inoculation. Shoot phenotyping over time, as here, revealed when growth depressions appeared in three different plant species, and it would be important to have a similar dataset for root biomass, as well as for plant nutrient concentrations over time.

## Supplementary Materials

Supplementary 1 Supplementary Table 1: ANOVA outcomes for three plant species for harvest time point data (mycorrhizal colonisation, dry biomass, and shoot nutrient concentrations); values in bold correspond to the selected terms. Supplementary Table 2: values for measures of plant biomass at harvest (treatment mean and standard error of the mean), in AMF-inoculated (+M) and noninoculated (-M) barley, tomato, and Medicago plants. Supplementary Table 3: for predicted PSA, summaries for three plant species of the *p*-values for the Wald F-statistics that test the effects associated with* Mycorrhiza* and* Zinc* treatments; values in bold correspond to the selected terms.* Supplementary Figure 1*: principal components analysis (PCA) biplot displaying scores in the first two principal components (PC1: x-axis; PC2: y-axis) following PCA of nine shoot elemental concentrations (black diamonds) in mock-inoculated (red symbols) and* R. irregularis*-inoculated (blue symbols) barley (a) and tomato (b) plants grown at Zn 0 (circles), Zn 10 (plus symbol), Zn 40 (diamonds), or Zn 90 (crosses), and Medicago (c) plants grown at Zn 0 (circles), Zn 2 (diamonds), Zn 5 (Y symbols), Zn 10 (plus symbols), Zn 20 (crosses), or Zn 30 (triangles). AMF treatment was included as a supplemental variable in the PCA (blue squares). The sign and magnitude of the contribution of elements are indicated by the arrows.

Supplementary 2 
* Supplementary Figure 2*: projected shoot area (PSA) over time grouped by* Mycorrhiza* treatment (+/- AMF inoculation with* R. irregularis*) in barley (a), tomato (b), and Medicago (c) plants grown at four (a, b) or six (c) different soil Zn concentrations ranging from no addition of Zn (Zn 0) to high soil Zn addition. On each panel, the darker lines represent the loess mean PSA of replicates within a treatment, while lighter lines correspond to individual replicates.

Supplementary 3 
* Supplementary Figure 3*: for barley* (H. vulgare)*, the predicted PSA for the main effect of* Mycorrhiza* inoculated with the AMF* R. irregularis *(blue) or mock-inoculated (red), grown at four different soil Zn concentrations from no addition of Zn to high soil Zn addition. The error bars are ±1/2 the LSD. Consequently, a pair of predictions whose error bars do not overlap is significantly different.
