# Supplementary material for: Using High-Throughput Phenotyping to Explore Growth Responses to Mycorrhizal Fungi and Zinc in Three Plant Species
Source: Plant Phenomics. 2019 Mar 25;2019:5893953. doi: 10.34133/2019/5893953 (PMC7718633; doi:10.34133/2019/5893953)
Supplement: Supplementary 1 — Supplementary Table 1: ANOVA outcomes for three plant species for harvest time point data (mycorrhizal colonisation, dry biomass, and shoot nutrient concentrations); values in bold correspond to the selected terms. Supplementary Table 2: values for measures of plant biomass at harvest (treatment mean and standard error of the mean), in AMF-inoculated (+M) and noninoculated (-M) barley, tomato, and Medicago plants. Supplementary Table 3: for predicted PSA, summaries for three plant species of the p-values for the Wald F-statistics that test the effects associated with Mycorrhiza and Zinc treatments; values in bold correspond to the selected terms. Supplementary Figure 1: principal components analysis (PCA) biplot displaying scores in the first two principal components (PC1: x-axis; PC2: y-axis) following PCA of nine shoot elemental concentrations (black diamonds) in mock-inoculated (red symbols) and R. irregularis-inoculated (blue symbols) barley (a) and tomato (b) plants grown at Zn 0 (circles), Zn 10 (plus symbol), Zn 40 (diamonds), or Zn 90 (crosses), and Medicago (c) plants grown at Zn 0 (circles), Zn 2 (diamonds), Zn 5 (Y symbols), Zn 10 (plus symbols), Zn 20 (crosses), or Zn 30 (triangles). AMF treatment was included as a supplemental variable in the PCA (blue squares). The sign and magnitude of the contribution of elements are indicated by the arrows. [file 5893953.f1.docx]

**Supplementary materials:**

**Tables**

Supplementary Table 1. ANOVA outcomes for three plant species for harvest time point data (mycorrhizal colonisation, dry biomass, shoot nutrient concentrations); values in bold correspond to the selected terms.

*Hordeum vulgare*

|  | AMF | Zn | AMF * Zn |
| --- | --- | --- | --- |
| Colonisation |  | **0.0135** |  |
| Shoot dry weight | **0.0003** | 0.5891 | 0.3611 |
| Root dry weight | **0.0003** | **0.0148** | 0.6037 |
| Root to shoot | 0.2915 | **0.0041** | 0.1146 |
| Shoot P conc. | 0.1743 | 0.4993 | 0.7627 |
| Shoot Zn conc. | <0.0001 | <0.0001 | **<0.0001** |

*Solanum lycopersicum*

|  | AMF | Zn | AMF * Zn |
| --- | --- | --- | --- |
| Colonisation |  | 0.2786 |  |
| Shoot dry weight | 0.0782 | <0.0001 | **0.0001** |
| Root dry weight | 0.0002 | <0.0001 | **0.0007** |
| Root to shoot | <0.0001 | 0.1217 | **0.0143** |
| Shoot P conc. | **<0.0001** | 0.7354 | 0.6243 |
| Shoot Zn conc. | <0.0001 | <0.0001 | **<0.0001** |

*Medicago truncatula*

|  | AMF | Zn | AMF * Zn |
| --- | --- | --- | --- |
| Colonisation |  | 0.0837 |  |
| Shoot dry weight | **0.0147** | **<0.0001** | 0.4106 |
| Root dry weight | **0.0458** | **<0.0001** | 0.0657 |
| Root to shoot | 0.0510 | **0.0006** | 0.0848 |
| Shoot P conc. | **<0.0001** | 0.1350 | 0.9169 |
| Shoot Zn conc. | 0.0128 | <0.0001 | **0.0001** |

|  | Zinc | Shoot dry weight (g) | | SEM | |  | Root dry weight (g) | | SEM | |  | Root to shoot ratio | | SEM | |
| --- | --- | --- | --- | --- | --- | --- | --- | --- | --- | --- | --- | --- | --- | --- | --- |
|  |  | -M | +M | -M | +M |  | -M | +M | -M | +M |  | -M | +M | -M | +M |
| *Hordeum vulgare* | Zn 0 | 0.561 | 0.370 | 0.052 | 0.014 |  | 0.999 | 0.677 | 0.131 | 0.075 |  | 1.78 | 1.82 | 0.20 | 0.15 |
|  | Zn 10 | 0.581 | 0.443 | 0.055 | 0.027 |  | 0.892 | 0.717 | 0.105 | 0.061 |  | 1.56 | 1.62 | 0.22 | 0.07 |
|  | Zn 40 | 0.565 | 0.431 | 0.037 | 0.052 |  | 0.703 | 0.542 | 0.070 | 0.052 |  | 1.26 | 1.28 | 0.16 | 0.07 |
|  | Zn 90 | 0.485 | 0.444 | 0.031 | 0.046 |  | 0.773 | 0.442 | 0.079 | 0.049 |  | 1.58 | 1.03 | 0.07 | 0.15 |
|  |  |  |  |  |  |  |  |  |  |  |  |  |  |  |  |
| *Solanum lycopersicum* | Zn 0 | 1.088 | 0.956 | 0.056 | 0.064 |  | 0.499 | 0.320 | 0.032 | 0.023 |  | 0.46 | 0.34 | 0.04 | 0.01 |
|  | Zn 10 | 1.064 | 1.151 | 0.051 | 0.125 |  | 0.477 | 0.372 | 0.014 | 0.024 |  | 0.45 | 0.33 | 0.03 | 0.03 |
|  | Zn 40 | 1.001 | 0.851 | 0.068 | 0.030 |  | 0.406 | 0.317 | 0.024 | 0.011 |  | 0.41 | 0.37 | 0.04 | 0.00 |
|  | Zn 90 | 0.360 | 0.909 | 0.019 | 0.057 |  | 0.228 | 0.290 | 0.035 | 0.018 |  | 0.62 | 0.32 | 0.07 | 0.01 |
|  |  |  |  |  |  |  |  |  |  |  |  |  |  |  |  |
| *Medicago truncatula* | Zn 0 | 2.561 | 2.443 | 0.121 | 0.160 |  | 4.313 | 2.210 | 0.617 | 0.189 |  | 1.64 | 0.91 | 0.20 | 0.06 |
|  | Zn 2 | 2.649 | 2.254 | 0.059 | 0.130 |  | 3.819 | 1.971 | 0.577 | 0.185 |  | 1.44 | 0.87 | 0.21 | 0.05 |
|  | Zn 5 | 2.507 | 2.581 | 0.201 | 0.145 |  | 2.674 | 4.203 | 0.371 | 0.936 |  | 1.04 | 1.61 | 0.07 | 0.33 |
|  | Zn 10 | 2.363 | 2.479 | 0.048 | 0.077 |  | 3.750 | 3.176 | 1.347 | 0.166 |  | 1.56 | 1.28 | 0.54 | 0.07 |
|  | Zn 20 | 2.130 | 1.816 | 0.080 | 0.086 |  | 2.561 | 2.091 | 0.391 | 0.491 |  | 1.20 | 1.12 | 0.19 | 0.22 |
|  | Zn 30 | 0.518 | 0.110 | 0.072 | 0.030 |  | 0.309 | 0.041 | 0.069 | 0.023 |  | 0.57 | 0.24 | 0.08 | 0.10 |

Supplementary Table 2. Values for measures of plant biomass at harvest (treatment mean and stardard error of the mean), in AMF-inoculated (+M) and non-inoculated (-M) barley, tomato, and Medicago plants.

Supplementary Table 3. For predicted PSA, summaries for three plant species of the $p$-values for the Wald F-statistics that test the effects associated with *Mycorrhiza* and *Zinc* treatments; values in bold correspond to the selected terms.

*Hordeum vulgare*

|  | AMF | Zn | AMF * Zn |
| --- | --- | --- | --- |
| Day 27 | 0.690 | 0.910 | 0.440 |
| Day 33 | 0.296 | 0.668 | 0.345 |
| Day 39 | **0.027** | 0.264 | 0.290 |
| Day 43 | **0.001** | 0.108 | 0.267 |
| Day 51 | 0.000 | 0.018 | 0.107 |

*Solanum lycopersicum*

|  | AMF | Zn | AMF * Zn |
| --- | --- | --- | --- |
| Day 27 | 0.329 | 0.000 | **0.004** |
| Day 33 | 0.022 | 0.000 | **0.000** |
| Day 39 | 0.006 | 0.000 | **0.000** |
| Day 43 | 0.042 | 0.000 | **0.000** |
| Day 51 | 0.519 | 0.000 | **0.004** |

*Medicago truncatula*

|  | AMF | Zn | AMF * Zn |
| --- | --- | --- | --- |
| Day 27 | 0.673 | 0.062 | 0.079 |
| Day 33 | 0.723 | 0.000 | **0.046** |
| Day 39 | 0.277 | 0.000 | **0.042** |
| Day 43 | 0.105 | 0.000 | **0.042** |
| Day 51 | **0.015** | **0.000** | 0.055 |

**Figures**


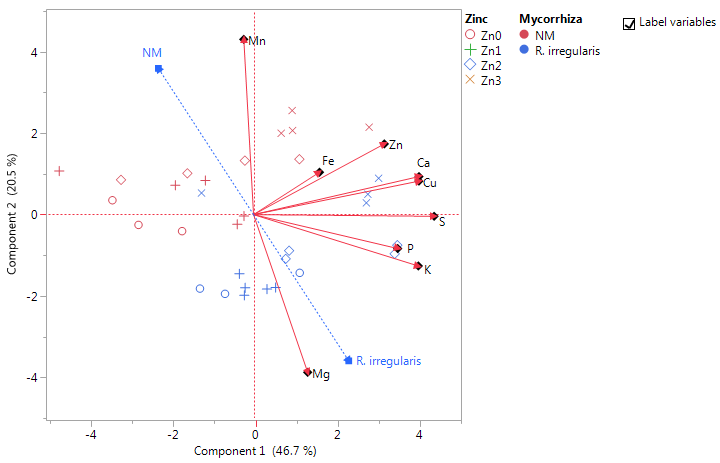

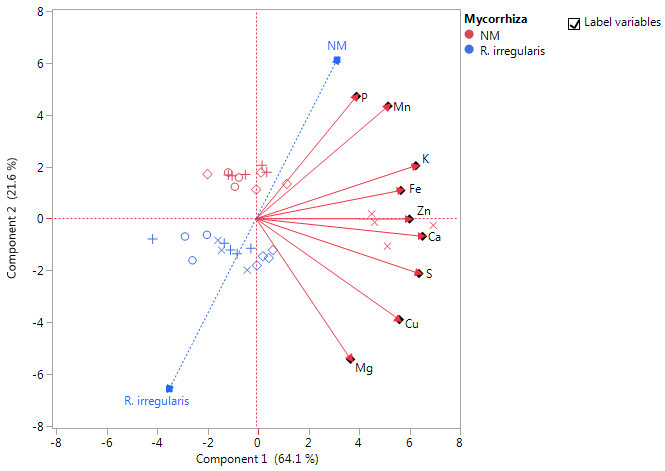


*Solanum lycopersicum*

*Hordeum vulgare*

b)

a)

*Medicago truncatula*

c)


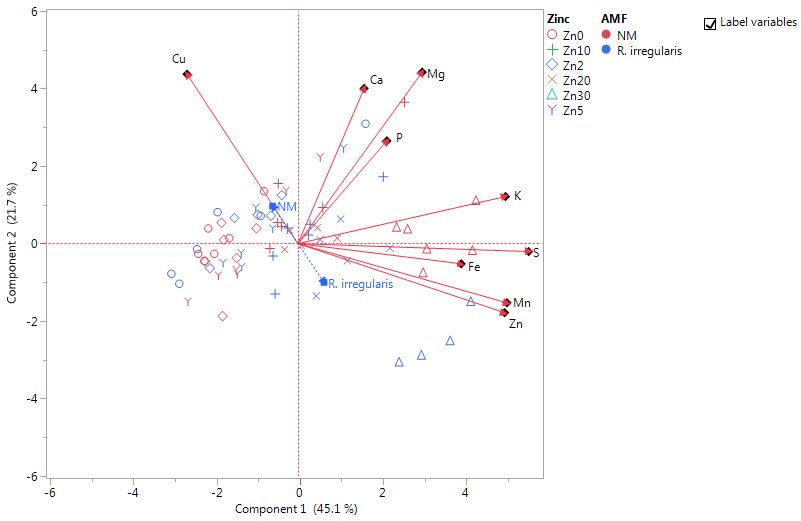


**Supplementary Figure 1.** Principal components analysis (PCA) biplot displaying scores in the first two principal components (PC1: x-axis, PC2: y-axis) following PCA of nine shoot elemental concentrations (black diamonds) in mock-inoculated (red symbols) and *R. irregularis*-inoculated (blue symbols) barley (a) and tomato (b) plants grown at Zn 0 (circles), Zn 10 (plus symbol), Zn 40 (diamonds) or Zn 90 (crosses), and Medicago (c) plants grown at Zn 0

c)

b)

a)
