# Supplementary figures and images for: Using High-Throughput Phenotyping to Explore Growth Responses to Mycorrhizal Fungi and Zinc in Three Plant Species
Source: Plant Phenomics. 2019 Mar 25;2019:5893953. doi: 10.34133/2019/5893953 (PMC7718633; doi:10.34133/2019/5893953)

### *Hordeum vulgare*

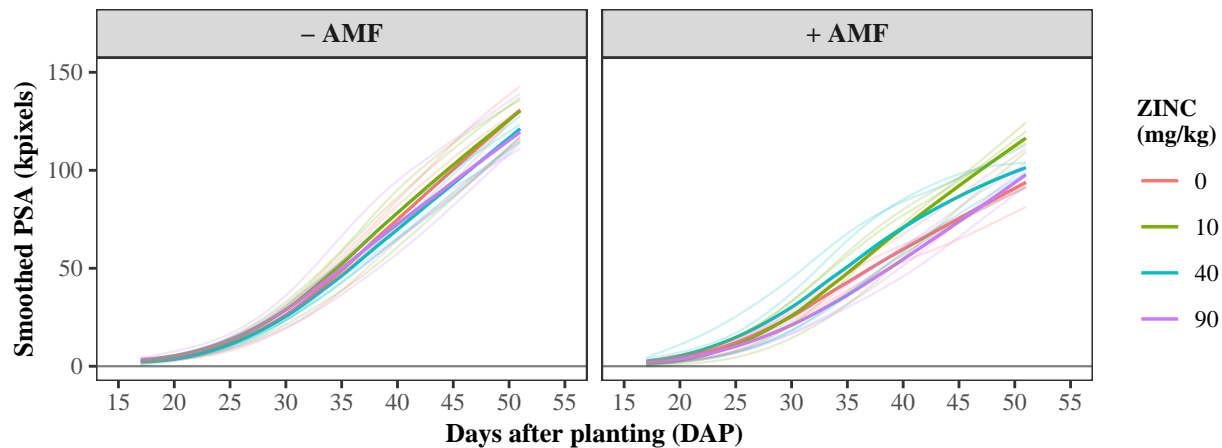

### *Solanum lycopersicum*

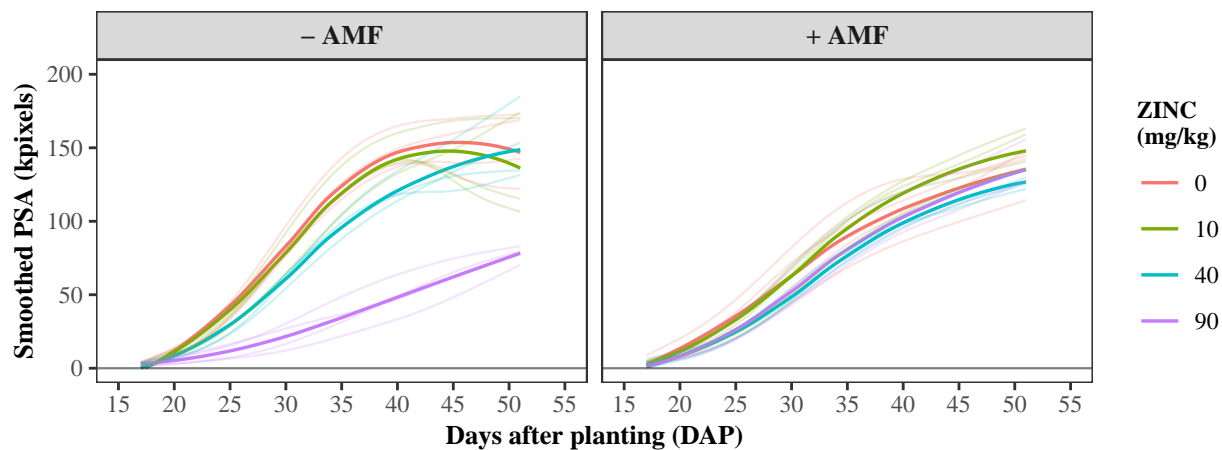

### *Medicago truncatula*

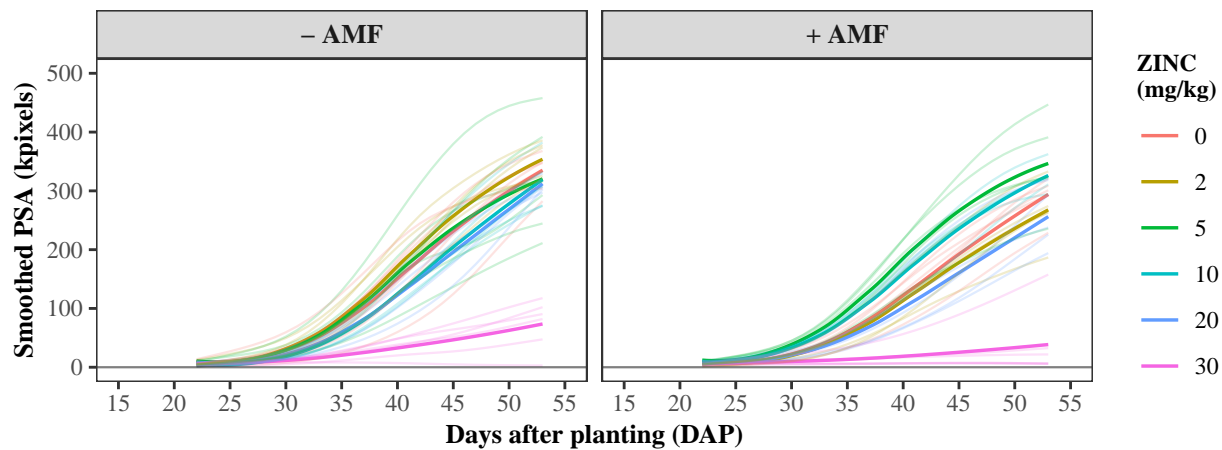

Supplement: Supplementary 2 — Supplementary Figure 2: projected shoot area (PSA) over time grouped by Mycorrhiza treatment (+/- AMF inoculation with R. irregularis) in barley (a), tomato (b), and Medicago (c) plants grown at four (a, b) or six (c) different soil Zn concentrations ranging from no addition of Zn (Zn 0) to high soil Zn addition. On each panel, the darker lines represent the loess mean PSA of replicates within a treatment, while lighter lines correspond to individual replicates. [file 5893953.f2.pdf]

*Hordeum vulgare*

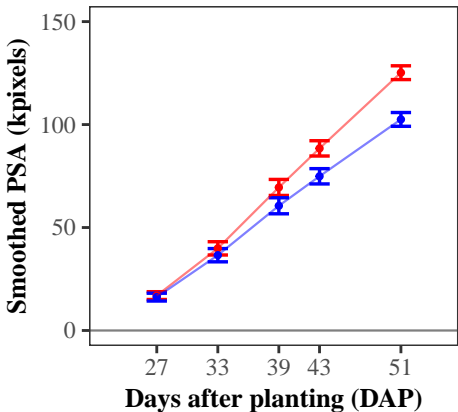

Supplement: Supplementary 3 — Supplementary Figure 3: for barley (H. vulgare), the predicted PSA for the main effect of Mycorrhiza inoculated with the AMF R. irregularis (blue) or mock-inoculated (red), grown at four different soil Zn concentrations from no addition of Zn to high soil Zn addition. The error bars are ±1/2 the LSD. Consequently, a pair of predictions whose error bars do not overlap is significantly different. [file 5893953.f3.pdf]
